# Binding Options for the Small Subunit-Like Domain of Cyanobacteria to Rubisco

**DOI:** 10.3389/fmicb.2020.00187

**Published:** 2020-02-28

**Authors:** Brandon A. Rohnke, Cheryl A. Kerfeld, Beronda L. Montgomery

**Affiliations:** ^1^MSU-DOE Plant Research Laboratory, Michigan State University, East Lansing, MI, United States; ^2^Department of Biochemistry and Molecular Biology, Michigan State University, East Lansing, MI, United States; ^3^Environmental Genomics and Systems Biology and Molecular Biophysics and Integrated Bioimaging Divisions, Lawrence Berkeley National Laboratory, Berkeley, CA, United States; ^4^Department of Microbiology & Molecular Genetics, Michigan State University, East Lansing, MI, United States

**Keywords:** photosynthesis, cyanobacteria, carboxysomes, rubisco, CcmM, carbon dioxide fixation

## Abstract

Two proteins found in cyanobacteria contain a C-terminal domain with homology to the small subunit of rubisco (RbcS). These small subunit-like domains (SSLDs) are important features of CcmM, a protein involved in the biogenesis of carboxysomes found in all β-cyanobacteria, and a rubisco activase homolog [activase-like protein of cyanobacteria (ALC)] found in over a third of sequenced cyanobacterial genomes. Interaction with rubisco is crucial to the function of CcmM and is believed to be important to ALC as well. In both cases, the SSLD aggregates rubisco, and this nucleation event may be important in regulating rubisco assembly and activity. Recently, two independent studies supported the conclusion that the SSLD of CcmM binds equatorially to L_8_S_8_ holoenzymes of rubisco rather than by displacing an RbcS, as its structural homology would suggest. We use sequence analysis and homology modeling to examine whether the SSLD from the ALC could bind the large subunit of rubisco either via an equatorial interaction or in an RbcS site, if available. We suggest that the SSLD from the ALC of *Fremyella diplosiphon* could bind either in a vacant RbcS site or equatorially. Our homology modeling takes into account N-terminal residues not represented in available cryo-electron microscopy structures that potentially contribute to the interface between the large subunit of rubisco (RbcL) and RbcS. Here, we suggest the perspective that binding site variability as a means of regulation is plausible and that the dynamic interaction between the RbcL, RbcS, and SSLDs may be important for carboxysome assembly and function.

## Introduction

Due to the evolution of a carbon concentrating mechanism (CCM), cyanobacteria are able to significantly contribute to global carbon fixation, despite the comparatively low atmospheric carbon dioxide (CO_2_) levels relative to their first appearance on Earth some 3.5 billion years ago (Schopf, 2012; Whitton and Potts, 2012). The CCM serves to significantly increase the flux of inorganic carbon into proteinaceous bacterial microcompartments called carboxysomes. Carboxysomes serve to encapsulate rubisco, and the shell acts as a semipermeable barrier to CO_2_ escape, allowing rubisco to function under high substrate levels (Dou et al., 2008). In the case of β-carboxysomes, which are present in cyanobacteria that express form 1B rubisco, synthesis occurs from the inside-out beginning with condensation of rubisco and carboxysomal protein CcmM into a liquid matrix (Cameron et al., 2013; Niederhuber et al., 2017; Wang et al., 2019).

The structure of CcmM is key to its nucleation of carboxysomal cargo. The C-terminus of CcmM contains three to five repeats of a domain that is homologous (around 60–70% similarity) to the small subunit of rubisco (RbcS)—denoted a small subunit-like domain (SSLD) (Price et al., 1993; Ludwig et al., 2000). The SSLD repeats domain-containing portion of CcmM can also be independently translated through an internal ribosome entry site. Both CcmM forms—full-length M58 and truncated M35—are necessary for normal carboxysome biogenesis (Long et al., 2010, 2011).

SSLDs were implicated in the interaction between rubisco and CcmM (Long et al., 2007; Cot et al., 2008) and were long hypothesized to bind in place of RbcS in rubisco complexes (Espie and Kimber, 2011; Long et al., 2011; Rae et al., 2013). However, recent structural work on SSLDs demonstrates equatorial binding of CcmM to L_8_S_8_ rubisco holoenzymes (Ryan et al., 2018; Wang et al., 2019). This binding appears to be driven largely by electrostatic interactions, and affinity for CcmM is not affected even when RbcS binding is partially compromised (Ryan et al., 2018).

SSLDs also appear as C-terminal domains in rubisco activase homologs [activase-like protein of cyanobacteria (ALC)] found in many cyanobacteria (Zarzycki et al., 2013). Recently, the ALC was shown to localize proximal to rubisco in the carboxysome and induce rubisco aggregation, much like M35 (Lechno-Yossef et al., 2019). Together, these findings provide strong evidence that the SSLD of ALC binds to rubisco and can induce coalescence of rubisco.

As recent findings indicate that SSLDs do not displace RbcS in L_8_S_8_ rubisco holoenzymes and instead bind equatorially, it is puzzling why there would be conservation of the RbcS-like secondary and tertiary structure elements that facilitate interactions with the large subunit of rubisco (RbcL). Some of the conserved residues from RbcS may fill repurposed roles in the SSLD-unique equatorial binding position, thus driving conservation of these features. Others, though, suggest that RbcS displacement may be possible. We decided to homology model the SSLD and RbcL and systematically compare the interfaces in order to evaluate the plausibility of equatorial versus RbcS substitution as a mode of binding (Ryan et al., 2018; Wang et al., 2019). We modeled the SSLD found in the ALC of *Fremyella diplosiphon* (*Fd*ALC SSLD) and analyzed the number and type of predicted interactions and free energy of solvation when the SSLD binds at the RbcS site (i.e., binds an empty site or displaces RbcS) or binds equatorially. We suggest that while equatorial binding was favored for CcmM in *Synechococcus elongatus* PCC 7942 (hereafter *Syn*7942), which lacks an ALC homolog, the *Fd*ALC SSLD had similar interface features in both positions. We propose that the *Fd*ALC could bind either equatorially or in place of RbcS and suggest that the current models of equatorial SSLD may be a part of a larger set of possibilities depending on specific proteins, for example, whether or not the cyanobacterium contains an ALC and perhaps is reflective of the recently uncovered diversity of cyanobacterial RbcL subunits (Lechno-Yossef et al., 2019).

## Materials and Methods

### Protein Homology Modeling

The structures of the *Fremyella* proteins were generated by homology modeling. For *Fd*ALC SSLD, the Swiss Model web server^1^ (Bertoni et al., 2017; Waterhouse et al., 2018) was used to generate a model for amino acid residues 317–424 based on *Syn*7942 CcmM SSLD1 in the reduced (PDB: 6HBB) form as well as *Synechococcus* sp. PCC 6301 (hereafter *Syn*6301) RbcS (PDB: 1RBL, Chain M). Additionally, a homology model of *Fremyella* L_8_S_8_ rubisco was made using *Syn*6301 rubisco (PDB: 1RBL) as a template. Alignment scores between two sequences were calculated using the LAlign webserver^2^ in order to evaluate candidate template structures and to compare primary structure conservation.

### Multiple Sequence Alignment of Activase-Like Protein of Cyanobacteria Small Subunit-Like Domains

The multiple sequence alignment (MSA) of 141 ALCs from cyanobacteria described in Lechno-Yossef et al. (2019) was trimmed to remove the ATPase domain, then the remaining regions (linker and SSLD) were realigned with a low gap cost at the end of sequences in CLC Sequence Viewer. This allowed for alignment of the SSLD region despite significant variations in the sizes of linkers between species, which were then trimmed to match the SSLD region identified in *Fd*ALC (residues 317–424, corresponding to residues 1–107 of the SSLD). An MSA was also generated for RbcS for each of the 128 organisms that had both a full-length SSLD and an annotated RbcS sequence. MSA for RbcS and SSLD were visualized and compared using HMM logos (Schuster-Böckler et al., 2004) generated on Skylign^3^.

### Analysis of Protein–Protein Interactions

**FIGURE 1:**
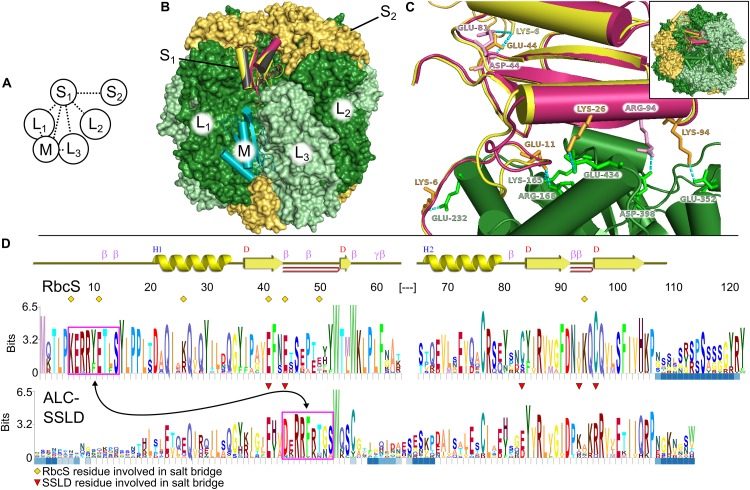
Structural comparison of small subunit of rubisco (RbcS) and small subunit-like domain (SSLD). **(A)** Schematic diagram of the interactions that a single RbcS (S_1_) or SSLD (M) has in a rubisco holoenzyme. Numbering based on van Lun et al. (2011). **(B)** Alignment of the SSLD found in activase-like proteins of cyanobacteria (ALCs) (black is based on an SSLD model, burgundy on an RbcS model) to the RbcS (yellow) or SSLD (teal) binding sites of an L_8_S_8_ rubisco holoenzyme. **(C)** Interaction between the large subunit of rubisco (RbcL_1_) (green) and RbcS (yellow) or SSLD (burgundy) in the context of the RubisCO holoenzyme. Labeled residues highlight residues predicted to interact in a salt bridge interaction (Supplementary Table S1), with label color based on the subunit it is from. Numbering for the SSLD is based on the trimmed region beginning at residue 317 in the full-length *Fd*ALC. Dashed cyan lines show the predicted interacting atoms. Inset indicates a zoomed-out view of the holoenzyme. **(D)** HMM-Logo highlighting the areas of sequence conservation between RbcS and the SSLD of ALC across cyanobacteria containing both. A schematic for the secondary structure of the homology model for RbcS from *Fremyella* is presented above the two logos. Blue squares below each residue depict regions with gaps in significant portions of the multiple sequence alignment (MSA). Magenta boxes, connected by an arrow, indicate a motif found in both MSAs. Yellow diamonds indicate the RbcS residues involved in salt bridge interactions in the *Fremyella* homology model, while red triangles indicate ALC-SSLD residues involved in salt bridge interactions in the S1 position in the homology model (Supplementary Table S1).

Using the homology model for L_8_S_8_ *Fremyella* rubisco, the two *Fd*ALC SSLD models were aligned to RbcS_1_ in PyMol, and structures were generated containing each SSLD replacing RbcS_1_. Another structure aligned *Syn*7942 CcmM SSLD1 in complex with rubisco (PDB: 6HBC) to the *Fremyella* rubisco, and then *Fd*ALC SSLD was aligned to the CcmM SSLD resulting in a *Fremyella* rubisco model with *Fd*ALC SSLD in the M position (Figure 1A). The *Syn*7942 CcmM SSLD1 structure was also used to replace the RbcS_1_ position in the *Syn*6301 rubisco L_8_S_8_ structure. Local refinement of structures was performed using Rosetta 3.4. Structures were subjected to the docking prepack protocol followed by the generation of 1,000 decoys using the docking protocol in docking local refine mode with the SSLD as the mobile target (Gray et al., 2003; Wang et al., 2005; Chaudhury et al., 2011). Based on interface score, the top 200 structures were clustered by pairwise RMSD with a 1 Å cutoff using energy-based clustering in Rosetta 3.4 (Hosseinzadeh et al., 2017). In all cases, the structure with the lowest interface score belonged to the largest cluster and was selected for use in downstream analysis.

These structures, as well as the *Fremyella* rubisco model and PDB: 6HBC, were analyzed using the Profunc web server^4^ (Laskowski, 2017). Interactions involving the RbcS and SSLD were compared for each structure. Further analyses were performed using the Pisa web server^5^ (Krissinel and Henrick, 2007) to calculate the solvation free energy gain (Δ*G*) upon formation of the interfaces for each structure.

## Binding at the RbcS_1_ Position

Each RbcS in an L_8_S_8_ assembly forms four unique protein–protein interfaces, three with the surrounding RbcL subunits and a fourth with a proximal RbcS (Figures 1A,B). The number of interfaces and predicted residue-level interactions were comparable to results from molecular dynamic simulations using *Chlamydomonas reinhardtii* rubisco (van Lun et al., 2011) and crystal structures (Knight et al., 1990). RbcS interactions with its nearest RbcL (RbcL_1_; Figure 1A) are substantial, burying ∼1,600 Å^2^ of surface area, with five predicted salt bridges, 13 hydrogen bonds and a free energy of solvation of −5.2 kcal⋅mol^–1^ (Table 1). The remaining interfaces bury less area and contain fewer bond interactions but favorably contribute to the overall L_8_S_8_ rubisco (Table 1, Column 5–*Fd*RbcS).

**TABLE 1:** Predicted small subunit interactions of rubisco from *Fremyella diplosiphon* and *Synechococcus elongatus* PCC 7942.

	Interacting Subunit	*Fd*ALC SSLD	*Fd*ALC SSLD (RbcS model)	*Fd*RbcS	*Syn*7942 CcmM SSLD1	*Syn*7942 CcmM SSLD1	*Fd*ALC SSLD
	
	Position	S_1_	S_1_	S_1_	S_1_	M	M
Number of Salt Bridges	L_1_	1	1	5	0	**1**	2
	L_2_	0	1	2	0	**0**	0
	L_3_	0	1	0	0	**1**	2
	S_1_	N/A	N/A	N/A	N/A	**1**	1
	S_2_	0	2	1	1	**0**	0
Number of Hydrogen Bonds	L_1_	2	9	13	1	**1**	4
	L_2_	0	4	6	1	**0**	0
	L_3_	1	2	1	0	**4**	3
	S_1_	N/A	N/A	N/A	N/A	**1**	2
	S_2_	4	2	3	3	**0**	0
Number of Non-bond Contacts	L_1_	17	108	194	7	**17**	34
	L_2_	2	26	77	7	**0**	0
	L_3_	16	29	44	10	**39**	21
	S_1_	N/A	N/A	N/A	N/A	**22**	21
	S_2_	19	12	20	20	**0**	0
Pisa Interface (Å^2^)	L_1_	380.3	1,326.5	1,591.5	218.5	**236.4**	448.9
	L_2_	91.0	495.1	614.9	260.0	**0.0**	0.0
	L_3_	313.2	359.4	467.9	232.4	**511.3**	479.4
	S_1_	N/A	N/A	N/A	N/A	**240.2**	239.3
	S_2_	173.5	240.7	261.4	249.6	**0.0**	0.0
Δ*G* (kcal⋅mol^–1^)	L_1_	0.9	1.3	−5.2	−0.6	−**1.4**	0.7
	L_2_	1.0	2.6	−1.0	0.3	**N/A**	N/A
	L_3_	−1.9	−1.5	−5.9	−1.0	**1.1**	3.1
	S_1_	N/A	N/A	N/A	N/A	**0.9**	−0.7
	S_2_	−3.1	−1.2	−3.2	−3.1	**N/A**	N/A

*Results from analyses using Profunc and Pisa web servers of protein–protein interactions in CcmM complexed with rubisco or a homology model of rubisco from *Fremyella*. Models contained homology models of *Fd*ALC small subunit-like domain (SSLD) or small subunit of rubisco (RbcS) from *Fremyella* or CcmM SSLD1 from *Syn*7942, as indicated by the row labeled “Interacting Subunit.” These subunits were aligned to the position indicated in row 2 (labels based on Figure 1A) in rubisco L_8_S_8_ structures from *Fremyella* (columns 3–5, 8), *Syn*6301 (column 6), or *Syn*7942 (column 7). Column 2 shows the rubisco subunit interface with the target SSLD/RbcS. Column 7, in bold, shows cryo-electron microscopy data from Wang et al. (2019).*

When RbcS is replaced with the SSLD-modeled *Fd*ALC SSLD, most salt bridges are lost (Table 1, Column 3–*Fd*ALC SSLD; Supplementary Table S1), as are many hydrogen bonds. This is particularly true at the L_1_-S_1_ interface, where the absence of the crucial N-terminal loop (residues 3–17) of RbcS in the SSLD accounts for three of the four missing salt bridges at this interface, as well as the significant reduction of major buried surface area (Knight et al., 1990; Ryan et al., 2018). SSLDs have two features that may play a role in this interaction. First, SSLDs have a poorly conserved flexible linker at their N-terminus that could be involved in non-specific interactions. Additionally, a portion of the N-terminal loop in RbcS involved in L_1_-S_1_ interactions is positionally displaced in the primary structures of SSLDs (Figure 1D, magenta box) (Ludwig et al., 2000). Notably, the structural position of this region corresponds to a helix in the SSLD structures but a loop in RbcS (Ryan et al., 2018; Wang et al., 2019). This “displaced motif” region is conserved and resembles the important lost motif of the N-terminus of RbcS but was not noted in Ryan et al. (2018) nor Wang et al. (2019) possibly because without significant backbone rearrangement, this motif is unlikely to be positioned to bind in the same way and its conservation could be attributed to its role in binding at the SSLD equatorial interface. Overall, our modeling with the truncated *Syn*CcmM SSLD template is consistent with the experimental observations that the SSLD structure has a significant loss of favorable binding interactions at the RbcS interface.

When the *Fd*ALC SSLD is modeled using RbcS as a template, part of the linker at the N-terminus of the SSLD (residues 1–17) is included in the model. Analysis of the *Fd*ALC SSLD in complex with rubisco suggested the potential for conservation of significantly more interactions [Table 1, Column 4–*Fd*ALC SSLD (RbcS model)]. Compared to the native RbcS, each interface buries slightly less area (∼80% of that observed for RbcS) and is predicted to contain fewer hydrogen bonds and non-bonding contacts. Many salt bridges are potentially maintained or are similar for a total of five compared to the eight found in RbcL–RbcS (Supplementary Table S1). For example, the SSLD model loses two salt bridges that contribute to the three structural checkpoints of the L_1_-S interface described in van Lun et al. (2011) (Figure 1C); this *Fd*ALC SSLD model is predicted to form a novel salt bridge with L_3_, and K6 forms an additional salt bridge with S_2_ instead of L_1_. Although the SSLD of ALCs shows many regions of relatively low conservation, the regions that are important for RbcS interactions are generally well conserved even in the SSLD (Figure 1D), with the notable uncertainty of the displaced N-terminal motif (magenta box) and linker. This suggests that when the flexible linker domain is also taken into account, *Fd*ALC SSLD could occupy an empty RbcS site.

## Binding at the Equatorial (M) Position

As reported in Wang et al. (2019), *Syn*CcmM SSLD1 forms favorable interactions with L_8_S_8_ rubisco in an equatorial position (we refer to this as position M). It forms a salt bridge with each rubisco subunit it contacts (L_1_, L_3_, and S_1_) and forms some hydrogen bonds (Table 1, Column 7–*Syn*7942 CcmM SSLD1). The Δ*G* at these three interfaces are less favorable overall than those calculated for the four interfaces of *Fd*ALC SSLD (Table 1, Column 3) and *Syn*CcmM SSLD1 in the S_1_ position (Table 1, Column 6–*Syn*7942 CcmM SSLD1). However, considering the presence of three salt bridges and that all RbcS positions were occupied in Ryan et al. (2018), these data are consistent with the model that SSLDs would bind equatorially rather than displace an engaged RbcS subunit.

*Fd*ALC SSLD also shows potential for interaction with the equatorial position, although the Δ*G* values calculated from these preliminary models are relatively less favorable. When bound at M, it is predicted to form a greater number of salt bridges and more hydrogen bonds compared to the *Syn*CcmM SSLD1 (Table 1, Column 8–*Fd*ALC SSLD).

## Discussion

Here, we present a prediction that the SSLDs found in cyanobacteria may be able to substitute for RbcS in binding RbcL; we propose that this could occur in addition to recently demonstrated equatorial binding (Ryan et al., 2018; Wang et al., 2019). Our analysis considers the SSLDs found in the absolutely conserved carboxysomal protein CcmM and the SSLDs found in the ALC, which is present in a subset of ecophysiologically diverse cyanobacteria. In the case of the *Fd*ALC SSLD, we found that its predicted binding with RbcL when substituting for the RbcS may be more favorable than that of the SSLD of CcmM of *Syn*7942, an organism that lacks the ALC. Although we find that the *Fd*ALC SSLD also could engage at the equatorial site, it may be a less favorable interaction than that observed for the SSLD of CcmM (Ryan et al., 2018; Wang et al., 2019). We suggest that the SSLD of *Fd*ALC has the potential for both equatorial binding and binding in the RbcS position. For *Syn*7942, the model organism used by Wang et al. (2019) and more closely related to *Thermosynechococcus elongatus* (Ryan et al., 2018), which also lacks an ALC, the predicted interface appears to point more favorably toward the equatorial binding found *in vitro*. Thus, it is possible that both the type of SSLD and the organism (i.e., class of RbcL) could influence whether the SSLD binds exclusively equatorially, especially for interactions with L_8_S_8_ rubisco holoenzymes. Additionally, in no case did we find that the SSLD bound better than the native RbcS, supporting the view that SSLDs cannot displace RbcS, though they might bind if sites are available.

In quantifications of protein abundance in cyanobacteria, a shortfall of RbcS relative to RbcL has been reported (Long et al., 2007, 2011; Sun et al., 2019). This finding suggests that isoforms other than the L_8_S_8_ rubisco holoenzyme may be present *in vivo*, although further substantiating analyses and investigations across multiple species as well as conditions are needed. Experiments in *Syn*7942 report five to six RbcS for eight RbcL, suggesting that vacant RbcS binding sites could be available *in vivo* (Long et al., 2011; Sun et al., 2019). Additionally, we suggest it may be possible that the RbcL:RbcS ratio could be dynamically regulated with impacts on both enzyme activity and the binding position of RbcS. Moreover, rubisco undergoes numerous posttranslational modifications that regulate its activity and subunit interactions (reviewed in Grabsztunowicz et al., 2017). In plants, phosphorylation influences rubisco kinetics (Lohrig et al., 2009) and modulate the interactions between RbcL, RbcS, and rubisco activase (Guitton and Mache, 1987; Aggarwal et al., 1993). Phosphorylation reversibly alters surface electrostatics potentially affecting the extent of equatorial SSLD interations. Notably, both the large and small subunits of cyanobacterial rubisco are targets of phosphorylation (Mikkat et al., 2014; Spät et al., 2015; Angeleri et al., 2016). These factors suggest mechanisms by which RbcS and SSLDs could be targets of dynamic regulation.

Throughout the evolution of diverse organisms containing Form I rubisco, a RbcL–RbcS fusion has never been observed. Given the importance of rubisco for survival, this is a significant clue to the potential for dynamic regulation of the RbcS:RbcL stoichiometry in cyanobacteria, potentially by the SSLDs. Though recent observations suggest that SSLDs bind primarily equatorially, we propose the possibility of a dynamic relationship between multiple binding locations. Such dynamics could play a large role in the nucleation of carboxysomes, which is fascinating given the observed impact that SSLD-containing proteins have on carboxysome morphology (Long et al., 2011; Rohnke et al., 2018). These features would seem to depend heavily on the availability of RbcS binding locations, the flexibility of the protein structures, redox state, posttranslational modifications, the species, the composition of the linker, the type of SSLD, and potentially even the subtype of RbcL (Lechno-Yossef et al., 2019), factors to consider in future investigations on the interaction(s) between rubisco and SSLDs.

## Data Availability Statement

The raw data supporting the conclusions of this article will be made available by the authors, without undue reservation, to any qualified researcher.

## Author Contributions

BR designed and conducted the research, analyzed and interpreted the data, and wrote the manuscript. CK and BM designed the research, analyzed and interpreted the data, and wrote the manuscript.

## Conflict of Interest

The authors declare that the research was conducted in the absence of any commercial or financial relationships that could be construed as a potential conflict of interest.

## Footnotes

1 https://swissmodel.expasy.org

2 https://embnet.vital-it.ch/software/LALIGN_form.html

3 http://skylign.org/

4 https://www.ebi.ac.uk/thornton-srv/databases/profunc/

5 https://www.ebi.ac.uk/msd-srv/prot_int/
