# Supplementary material for: Binding Options for the Small Subunit-Like Domain of Cyanobacteria to Rubisco
Source: Front Microbiol. 2020 Feb 28;11:187. doi: 10.3389/fmicb.2020.00187 (PMC7059596; doi:10.3389/fmicb.2020.00187)
Supplement: Supplementary file 1 [file Data_Sheet_1.docx]

**Table S1.** **Comparison of predicted salt bridges between RbcL and RbcS or the SSLD.** Hypothetical salt bridges in the *Fremyella* rubisco homology model interacting with either RbcS or *Fd*ALC SSLD models at position S_1_ (see Figure 1A). The SSLD from *Fd*ALC was modeled using either RbcS from *Syn*6301 as a template or the CcmM SSLD1 from *Syn*7942 as the template. Residue numbering corresponds to *Fremyella* sequences, with the SSLD corresponding to residues 317-424 of *Fd*ALC (e.g., residue R94 of the SSLD corresponds to residue R410 of *Fd*ALC).

| Interaction | Rubisco:RbcS1 | Rubisco:SSLD (RbcS Modeled) | RbcL:SSLD  (ALC SSLD Modeled) |
| --- | --- | --- | --- |
| L_1_-S_1_ | K165:E11 | - | - |
|  | R168:E11 | - | - |
|  | E232:K6 | - | - |
|  | E352:K94 | D398:R94 | D397:R94 |
|  | E434:K26 | - | - |
| L_2_-S_1_ | R188:E41 | R188:E41 | - |
|  | K228:E50 | - | - |
| L_3_-S_1_ | - | D77:K91 | - |
| S_2_-S_1_ | K6:E44 | K6:D44 | - |
|  | - | K6:E81 | - |
